# Limited Awareness and Low Immediate Uptake of Pre-Exposure Prophylaxis among Men Who Have Sex with Men Using an Internet Social Networking Site

**DOI:** 10.1371/journal.pone.0033119

**Published:** 2012-03-28

**Authors:** Douglas S. Krakower, Matthew J. Mimiaga, Joshua G. Rosenberger, David S. Novak, Jennifer A. Mitty, Jaclyn M. White, Kenneth H. Mayer

**Affiliations:** 1 Harvard Medical School, Boston, Massachusetts, United States of America; 2 Beth Israel Deaconess Medical Center, Boston, Massachusetts, United States of America; 3 Department of Psychiatry, Massachusetts General Hospital, Boston, Massachusetts, United States of America; 4 Department of Epidemiology, Harvard School of Public Health, Boston, Massachusetts, United States of America; 5 Department of Global and Community Health, George Mason University, Fairfax, Virginia, United States of America; 6 OLB Research Institute, Online Buddies, Inc., Cambridge, Massachusetts, United States of America; 7 The Fenway Institute, Fenway Health, Boston, Massachusetts, United States of America; Vanderbilt University, United States of America

## Abstract

**Background:**

In 2010, the iPrEx trial demonstrated that oral antiretroviral pre-exposure prophylaxis (PrEP) reduced the risk of HIV acquisition among high-risk men who have sex with men (MSM). The impact of iPrEx on PrEP knowledge and actual use among at-risk MSM is unknown. Online surveys were conducted to assess PrEP awareness, interest and experience among at-risk MSM before and after iPrEx, and to determine demographic and behavioral factors associated with these measures.

**Methods and Findings:**

Cross-sectional, national, internet-based surveys were administered to U.S. based members of the most popular American MSM social networking site 2 months before (n = 398) and 1 month after (n = 4 558) publication of iPrEx results. Comparisons were made between these samples with regards to PrEP knowledge, interest, and experience. Data were collected on demographics, sexual risk, and experience with post-exposure prophylaxis (PEP). Regression analyses were performed to identify factors associated with PrEP awareness, interest, and experience post-iPrEx. Most participants were white, educated, and indicated high-risk sexual behaviors. Awareness of PrEP was limited pre- and post-iPrEx (13% vs. 19%), whereas interest levels after being provided with a description of PrEP remained high (76% vs. 79%). PrEP use remained uncommon (0.7% vs. 0.9%). PrEP use was associated with PEP awareness (OR 7.46; CI 1.52–36.6) and PEP experience (OR 34.2; CI 13.3–88.4). PrEP interest was associated with older age (OR 1.01; CI 1.00–1.02), unprotected anal intercourse with ≥1 male partner in the prior 3 months (OR 1.40; CI 1.10–1.77), and perceiving oneself at increased risk for HIV acquisition (OR 1.20; CI 1.13–1.27).

**Conclusions:**

Among MSM engaged in online networking, awareness of PrEP was limited 1 month after the iPrEx data were released. Utilization was low, although some MSM who reported high-risk behaviors were interested in using PrEP. Studies are needed to understand barriers to PrEP utilization by at-risk MSM.

## Introduction

As 61% of new HIV infections in the United States occur among men who have sex with men (MSM) [1], new prevention strategies are urgently needed in this population. In November 2010, the iPrEx study demonstrated that oral antiretroviral pre-exposure chemoprophylaxis (PrEP) with a once-daily tablet containing a fixed dose combination of tenofovir disoproxil fumarate and emtricitabine (FTC-TDF) reduced the risk of HIV acquisition among at-risk MSM and transgendered women compared to a placebo-control [2]. These clinical trial results have raised numerous questions about how to optimize public health benefits of this strategy in real-world settings, such as how to increase PrEP adherence and cost effectiveness and prevent risk compensation, toxicities, and the emergence of drug resistance [3]. Though the role of oral PrEP in prevention efforts may evolve as new data emerge, a prerequisite for any degree of implementation will be identifying and engaging individuals who are most likely to benefit from PrEP use [4].

Several years before publication of the iPrEx results, convenience surveys at gay pride events in several United States cities suggested PrEP awareness (25%) and use (5%) among MSM were relatively low [5]. Subsequent cross-sectional surveys of MSM in California and New England corroborated that PrEP awareness was modest (16–19%) and experience was rare (<1%) [6], [7], though the men were amenable to consider using it if PrEP was shown to be efficacious (67–74%) [6], [7]. Because FTC-TDF is FDA-approved, this high degree of hypothetical interest suggested that it was feasible that a rapid increase in MSM demand for oral PrEP could occur after release of efficacy data. To prepare for this, the U.S. Centers for Disease Control and Prevention issued an interim guidance on PrEP for MSM within two months of the publication of the iPrEx results [8]. However, the actual impact of these findings for MSM has been unclear.

To assess for changes in PrEP awareness, interest, and use after the release of efficacy results, repeated cross-sectional, national, online surveys of MSM using an internet social networking site were administered two months before and one month after the publication of iPrEx results. In addition, demographic and behavioral factors associated with PrEP awareness, interest, and experience post-iPrEx were determined to identify subgroups that may be potential targets for educational interventions. As a substantial proportion of MSM seek sexual partners online [9], [10], and MSM who engage in online sexual networking may be at high risk for HIV acquisition [11], [12], [13], [14], surveys were administered through a popular sexual partner-seeking/social networking website for MSM. Since online sites could provide an attractive avenue for disseminating information about PrEP [10], [15], it was particularly important to study community norms in this setting.

## Methods

### Ethics Statement

The Institutional Review Boards of The Fenway Institute and Beth Israel Deaconess Medical Center approved the study procedures. All participants provided internet-based informed consent. As all data were analyzed anonymously, documentation of written informed consent was waived by these Institutional Review Boards.

### Participants and Procedures

Members of a large, multinational social networking site for MSM were invited to complete an anonymous online survey about PrEP knowledge, interest and experience before (September–October, 2010) and after (December–January, 2011) the release of iPrEx trial data. At the start of the computer interview, a general description of PrEP was provided to pre- and post-iPrEx groups. After this description, data were collected on PrEP awareness, interest, and experience. For the purpose of these analyses, iPrEx results were not provided, since the goal was to capture an unbiased assessment of community norms after public reporting of iPrEx. Pre-iPrEx, the survey contained 74 items and required approximately 15–20 minutes to complete. Post-iPrEx, 11 additional items (not included in the current analyses) were added to the end of the survey, extending the survey by approximately 4 minutes. Participants were eligible if they were biologically male at birth, at least 18 years of age, HIV-uninfected by self-report, and could read and understand English and use computers and the Internet.

Pre-iPrEx, an email broadcast was sent to the most active United States members (n = 20 000) with an invitation to learn about the study: 13 284 received and opened the emails, 1 790 (13.5%) clicked through to the survey, 581 (32.5%) consented to complete a pre-screening questionnaire, 473 (81.4%) met eligibility criteria, and 398 (84.1%) consented to enroll in the study and have their data analyzed, representing a 22.2% response rate of those who clicked on the hyperlink for the study; 134 (33.7%) of participants abandoned the study before completion. Post-iPrEx, another email broadcast was sent with an invitation to learn about the study, and the number of members targeted was increased to include all U.S. members (excluding those with self-disclosed HIV infection) given modest enrollment rates from the pre-iPrEx phase: 93 972 members received and opened the emails, 16 715 (17.8%) clicked through to the survey, 6 267 (37.5%) consented to complete a pre-screening questionnaire, 5 399 (86.1%) met eligibility criteria, and 4 558 (84.4%) consented to enroll in the study and have their data analyzed, representing a 27.3% response rate; 1 584 (34.8%) of participants abandoned the study before completion. For participants who did not complete the full survey, data were analyzed for all questions that were answered. As such, the total number of respondents is given for each of the measures reported. Thirty-seven U.S. states and the District of Columbia were represented pre-iPrEx, and all 50 states and the District of Columbia were represented post-iPrEx.

### Measures

#### Demographics

Participants reported information on demographics and history of sexual partnerships [16].

#### Psychosocial Factors

To screen for alcoholism, the 4-item CAGE questionnaire was administered [17], [18], [19]. A score of ≥10 on the 10-item version of the Centers for Epidemiologic Studies Depression (CES-D) scale, a validated survey to assess for depressive symptoms, was considered a positive screen for depression [20].

#### Sexual Risk

Data were collected on prior sexually transmitted infections (STI) and high-risk sexual behaviors, including recreational substance use during sex and unprotected anal intercourse with multiple partners or partners who are reportedly HIV-infected or of unknown HIV serostatus.

#### Self-Perceived Risk of HIV Acquisition

Participants rated their risk on a scale of 1 (“Not risky at all”) to 10 (“Extremely risky”) based on their sexual behaviors in the prior 3 months [21], [22].

#### Engagement in Healthcare

Participants were asked if they had visited a healthcare provider in the prior 12 months and whether they identified a primary care provider (PCP) [23].

#### Communication with Providers

To assess communication about HIV risk behaviors, participants were asked to indicate their level of comfort discussing same-sex behaviors with a PCP on a 5-point scale from 1 (“Extremely uncomfortable”) to 5 (“Extremely comfortable”); responses were dichotomized for analyses such that a rating of ≤3 was categorized as “Not comfortable” and ≥4 was “Comfortable” to provide a conservative estimate of comfort levels. Participants were also asked if they had discussed unprotected anal sex behaviors or ways to protect themselves against HIV infection with a PCP, based on a prior study focused on patient-provider communication among MSM [23].

#### Post-Exposure Prophylaxis (PEP)

Participants were asked about their awareness and experience with PEP based on questions employed in previous studies conducted at the Fenway Institute [7], [24].

#### PrEP Awareness, Interest, and Experience

Data were collected on PrEP awareness, interest, and experience using questions adapted from a prior study of PrEP attitudes among MSM [7].

### Data Analyses

OpenEpi (Atlanta, GA) [25] was used to perform t-tests and χ^2^ tests of independence, and SAS version 9.2 (Cary, NC) was used for descriptive statistics and logistic regression modeling. Tests were statistically significant at the P<0.05 level. Minimal non-independence of respondents contributing to pre- and post-iPrEx samples was presumed given the large difference in sample sizes. Because data were obtained using different recruitment methods for each sample, descriptive analyses were used instead of formal statistical testing to compare pre- and post-iPrEx results. The characteristics of participants who completed the survey pre- and post-iPrEx were stratified by awareness of PrEP. To determine the relationship between participant characteristics and outcomes of interest, bivariate logistic regression procedures were conducted. Variables significant at P<0.05 were included in three distinct multivariable models to determine factors that were independently associated with PrEP awareness, interest, and experience. Multicollinearity was assessed among independent variables. None of the independent variables were found to be intercorrelated at or above a threshold of 0.80 and all were retained in the final models. Regression procedures were conducted only on post-iPrEx data, as the substantially larger sample size was more likely to result in meaningful analyses.

## Results

### Participant Sociodemographic and Behavioral Characteristics

Demographics of MSM who participated in the study pre- and post-iPrEx are presented in Table 1. The majority of participants pre- and post-iPrEx were white, highly educated, and employed full-time, with over one-third of MSM reporting annual income of $60 000 or more. Nearly all men self-identified as gay or bisexual (>99%). About a quarter of the men had a clinical history of depression and almost 1/5 screened in for depression when they filled out the survey. Prior drug or alcohol treatment, and current alcohol abuse, were also common (Table 1). Over half of the pre-iPrEx participants reported unprotected anal intercourse (UAI) with at least one male partner of any HIV serostatus in the 3 months prior to completing the survey, and approximately one-quarter reported UAI with at least one male partner of reportedly HIV-positive or unknown serostatus or a history of STI. The men who filled out the survey post-iPrEx were comparably risky, but a smaller percentage of the respondents indicated they had engaged in UAI while using recreational drugs. Most participants perceived themselves to be at low to moderate risk to acquire HIV. Overall, no clinically meaningful differences in participant sociodemographic and behavioral characteristics were apparent between the samples.

**Table 1:** Sociodemographic and Behavioral Characteristics of Study Participants Two Months before (n = 398) versus One Month after (n = 4 558) Publication of Oral Pre-Exposure Prophylaxis Efficacy Results (the iPrEx Study).

		Pre-iPrEx (N = 398)		Post-iPrEx (N = 4558)	
		*Mean (SD)*	*N* 1	*Mean (SD)*	*N* 1
Age		40.2 (12.1)	398	39.0 (12.8)	4558
Number of male partners (UAI) in prior 3 mo.		2.4 (5.5)	395	2.1 (5.3)	4558
Self-perceived risk of HIV acquisition^2^		3.6 (2.4)	288	3.3 (2.3)	3739
		***%***		***%***	
Pre-Exposure Prophylaxis (PrEP)	Aware of PrEP	12.5	289	19.0	3387
	Interested in using PrEP^3^	76.1	289	78.5	3382
	Have used PrEP	0.7	289	0.9	3385
Sexual Orientation^4^	Homosexual or Gay	88.8	268	83.2	2977
	Bisexual	11.2	268	16.3	2977
Gender of Sex Partners - prior 3 mo.	Men Only	95.9	291	92.6	3401
	Men and Women	4.1	291	7.4	3401
Race/Ethnicity	Asian/Asian American/Pacific Islander	6.0	268	2.7	3003
	Hispanic/Latino	7.1	268	5.9	3003
	African American/Black	2.2	268	3.2	3003
	White	82.1	268	84.0	3003
	Native American/Alaskan Native	0.4	268	0.6	3003
	Multiracial/Other	2.2	268	3.6	3003
Education	≤ High school/GED	6.4	266	6.6	3003
	Some College - College graduate	59.8	266	56.7	3003
	≥ Some Graduate training	32.8	266	36.8	3003
Employment	Full-Time (≥30 hrs./wk.)	63.3	267	67.0	3001
	< Full-time, Student, Other	36.7	267	33.0	3001
Annual Income (Pre-tax)	≤$17,999	16.7	263	18.5	2958
	$18,000–$29,999	16.3	263	13.0	2958
	$30,000–$59,999	25.5	263	29.4	2958
	≥$60,000	41.4	263	39.1	2958
Health Insurance - Covered		83.1	255	85.8	2892
High-Risk Sex - prior 3 mo.	UAI with ≥1 male partner	58.4	397	61.8	4558
	UAI with ≥5 male partners	12.2	395	10.4	4558
	UAI with ≥1 HIV-infected or unknown serostatus male partner	24.2	393	23.4	4555
	Transactional sex	6.2	292	7.3	3469
	UAI after ≥5 drinks	24.3	263	23.0	2975
	UAI while using recreational drugs	18.6	264	10.9	2974
Prior STI		27.7	398	28.0	4558
Post-Exposure Prophylaxis (PEP)	Heard of PEP	38.1	289	36.2	3396
	Used PEP	4.5	289	3.7	3394
Engagement in Healthcare	Contact w/any provider - prior 12 mo.	85.9	269	89.1	3113
	Identifies a PCP	76.6	269	81.8	3113
Communication with PCP	PCP aware of UAI behaviors^5^	47.5	143	38.2	1666
	Comfortable discussing same-sex behaviors w/PCP	68.8	205	61.5	2537
	Discussed ways to protect against HIV w/PCP	47.8	205	43.7	2536
Psychosocial Factors	History of depression	24.7	267	25.3	2988
	Positive screen - depression (CES-D)	31.9	263	25.5	2956
	Ever treated for drug or alcohol abuse	3.4	263	4.8	2970
	Positive screen - alcohol abuse (CAGE)	18.4	261	16.3	2965

UAI = unprotected anal intercourse; STI = sexually transmitted infection; PCP = primary care provider; CES-D = Center for Epidemiologic Studies Depression Scale; CAGE = 4-question screen for alcohol abuse.

1 Total number of participants responding to each question.

2 Self-perceived risk of HIV acquisition = scale from 1 (no risk) to 10 (extreme risk).

3 Interested in using PrEP = likely or extremely likely to use PrEP.

4 Excludes 15/2977 (0.5%) participants who self-identified as heterosexual post-iPrEx.

5 Among participants indicating UAI in the prior 3 months.

### Engagement in Healthcare, Communication with Providers, and Post-Exposure Prophylaxis Experience

Most participants reported contact with a healthcare provider in the prior 12 months and identified a primary care provider (Table 1). Less than half of MSM had discussed ways to protect against HIV with a PCP. Although approximately one-third of participants were aware of PEP in each group, prior use was uncommon (Table 1).

### PrEP Awareness and Interest

Awareness of PrEP was limited but increased after iPrEx (12.5% (36/289) pre-iPrEx vs. 19.0% (642/3387) post-iPrEx). Fewer participants of moderate income ($18 000–$29 000 per year) were aware of PrEP after iPrEx (25.0% pre-iPrEx vs. 10.5% post-iPrEx). Otherwise, MSM who were aware of PrEP pre- and post-iPrEx did not appear to differ sociodemographically or behaviorally. Pre- and post-iPrEx, the majority of the participants expressed interest in using PrEP after they were provided with a brief description about it (76.1% (220/289) pre-iPrEx vs. 78.5% (2654/3382) post-iPrEx).

In multivariable analysis, being aware of PrEP was associated with identifying as bisexual compared to gay (OR 1.87; 1.01–3.46; p = 0.05), awareness of PEP versus no awareness (OR 33.7; CI 21.3–53.3; p<0.0001), and prior PEP use compared to never having used PEP (OR 1.97; CI 1.15–3.38; p = 0.01) (Table 2).

**Table 2:** Participant Characteristics associated with Awareness of Pre-Exposure Prophylaxis (n = 642) One Month after Publication of Efficacy Results (the iPrEx Study).

		Aware of PrEP					
		Bivariate OR	95% CI	P	Multivariable OR	95% CI	P
Sexual Orientation	Homosexual or Gay	1.00	-	-	1.00	-	-
	Bisexual	0.50	0.37–0.67	<0.0001	1.87	1.01–3.46	0.05
Post-exposure Prophylaxis (PEP)	Aware of PEP	38.4	28.5–51.8	<0.0001	33.7	21.3–53.3	<0.0001
	Not aware of PEP	1.00	-	-	1.00	-	-
	Prior PEP use	5.21	3.63–7.49	<0.0001	1.97	1.15–3.38	0.01
	No prior PEP use	1.00	-	-	1.00	-	-

PrEP = pre-exposure prophylaxis; OR = odds ratio; CI = Confidence Interval; P = level of significance.

Variables that were not statistically significant in bivariate analyses and were not entered into the multivariable model include: number of male partners for unprotected anal intercourse (UAI) in the prior 3 months; self-perceived risk of HIV acquisition; monogamous status; race/ethnicity; health insurance coverage; UAI with ≥1 male partner, UAI with ≥5 male partners, UAI with ≥1 male partner who is HIV-infected or of unknown serostatus, transactional sex, UAI after ≥5 drinks, and UAI while using recreational drugs, each over the prior 3 months; identification of a primary care provider; diagnostic history of clinical depression; positive screen for depressive symptoms (Center for Epidemiologic Studies Scale); and prior treatment for drug or alcohol abuse. Variables that were not statistically significant in multivariable analyses include: interest in PrEP (likely or extremely likely to use PrEP); gender of sexual partners in prior 3 months; educational attainment; employment status; annual income; history of sexually transmitted infection; contact with any healthcare provider in the prior 12 months; among participants indicating UAI in the prior 3 months, having a primary care provider (PCP) who is aware of UAI behaviors; comfort discussing same-sex behaviors with PCP; having discussed ways to protect against HIV acquisition with PCP; and positive screen for alcohol abuse (4-item CAGE questionnaire).

In a separate multivariable model, interest in PrEP use was associated with being older (OR 1.01; CI 1.00–1.02; p = 0.01), having greater self-perceived risk of HIV acquisition (OR 1.20; CI 1.13–1.27; p<0.0001), and UAI with at least 1 male partner in the prior 3 months versus no UAI in the prior 3 months (OR 1.41; CI 1.11–1.79; p = 0.004); awareness of PEP, compared to no knowledge of PEP, was associated with decreased interest in using PrEP (OR 0.55; CI 0.43–0.71; p<0.0001) (Table 3).

**Table 3:** Participant Characteristics associated with Interest in Using Pre-Exposure Prophylaxis (n = 2 654) One Month after Publication of Efficacy Results (the iPrEx Study).

		Interested in Using PrEP^1^					
		Bivariate OR	95% CI	P	Multivariable OR	95% CI	P
Age		1.01	1.01–1.02	0.0002	1.01	1.00–1.02	0.01
Self-perceived risk of HIV acquisition^2^		1.20	1.15–1.25	<0.0001	1.20	1.13–1.27	<0.0001
High-Risk Sex - prior 3 mo.	UAI with ≥1 male partner	1.72	1.45–2.03	<0.0001	1.41	1.11–1.79	0.004
	No UAI with male partner	1.00	-	-	1.00	-	-
Post-Exposure Prophylaxis (PEP)	Aware of PEP	0.51	0.44–0.61	<0.0001	0.55	0.43–0.71	<0.0001
	Not aware of PEP	1.00	-	-	1.00	-	-

PrEP = pre-exposure prophylaxis; OR = odds ratio; CI = Confidence Interval; P = level of significance; UAI = unprotected anal intercourse.

1 Interested in Using PrEP = likely or extremely likely to use PrEP.

2 Self-perceived risk of HIV acquisition = scale from 1 (no risk) to 10 (extreme risk).

Variables that were not statistically significant in bivariate analyses and were not entered into the multivariable model include: monogamous status; race/ethnicity; employment status; annual income; health insurance coverage; transactional sex, UAI after ≥5 drinks and UAI while using recreational drugs - each over the prior 3 months; history of sexually transmitted infection; prior PEP use; contact with any healthcare provider in prior 12 months; identification of a primary care provider (PCP); among participants indicating UAI in prior 3 months, having a PCP who is aware of UAI behaviors; diagnostic history of clinical depression; positive screen for depressive symptoms (Center for Epidemiologic Studies Scale); and prior treatment for drug or alcohol abuse. Variables that were not statistically significant in multivariable analyses include: number of male partners for UAI in the prior 3 months; awareness of PrEP; sexual orientation; gender of sexual partners in prior 3 months; educational attainment; UAI with ≥5 male partners and UAI with ≥1 male partner who is HIV-infected or of unknown serostatus - each over the prior 3 months; comfort discussing same-sex behaviors with PCP; having discussed ways to protect against HIV acquisition with PCP; and positive screen for alcohol abuse (4-item CAGE questionnaire).

### PrEP Experience

Pre-iPrEx, 0.7% (2/289) of respondents reported having used PrEP, whereas post-iPrEx, 0.9% (29/3385) did. The sources of PrEP for the 29 individuals who reported prior PrEP use included: their PCP (8); another healthcare provider (8); a friend (6); the internet (1); a sex partner (2); and “Other” (8) without further details. Three respondents indicated participation in a clinical trial of PrEP. Eighty-percent (20/25) reported using PrEP on a daily basis for a period of time, while the others used PrEP only right before sex. Compared to MSM who had not used PrEP, a greater percentage of those with PrEP experience reported UAI with at least one male partner, and had at least 5 male partners in the prior 3 months. PrEP users were more likely to know about, and have used, PEP and to have engaged in discussions with a PCP about ways to protect themselves against HIV (Table 4).

**Table 4:** Characteristics of Participants Reporting Use of Pre-Exposure Prophylaxis (n = 29) compared to Non-Users (n = 3 356) One Month after Publication of Efficacy Results (the iPrEx Study).

		Have used PrEP (n = 29)		Have not used PrEP (n = 3356)		
		*Mean (SD)*	*N* 1	*Mean (SD)*	*N* 1	
Age		41.2 (11.3)	29	39.7 (12.8)	3356	
Number of male partners (UAI) - prior 3 mo.		4.7 (9.4)	29	2.3 (5.0)	3356	
Self-perceived risk of HIV acquisition^2^		4.0 (2.2)	29	3.3 (2.3)	3353	
		***%***		***%***		
Pre-Exposure Prophylaxis (PrEP)	Interested in using PrEP^3^	77.8	27	78.5	3354	
Sexual Orientation	Homosexual or Gay	69.6	23	83.7	2937	
	Bisexual	30.4	23	16.3	2937	
Gender of Sex Partners - prior 3 mo.	Men only	82.1	28	92.7	3286	
	Men and Women	17.9	28	7.3	3286	
Race/Ethnicity	White	78.3	23	84.1	2978	
	Other	21.7	23	15.9	2978	
Education	≤ High school/GED	4.4	23	6.6	2978	
	Some college - College graduate	52.2	23	56.7	2978	
	≥ Some graduate training	43.5	23	36.7	2978	
Employment	Full-time (≥30 hrs./wk.)	65.2	23	67.0	2976	
	< Full-time, Student, Other	34.8	23	33.0	2976	
Annual Income (Pre-tax)	≤$17,999	21.7	23	18.5	2933	
	$18,000–$29,999	13.0	23	13.1	2933	
	$30,000–$59,999	21.7	23	29.4	2933	
	≥$60,000	43.5	23	39.0	2933	
Health Insurance – Covered		95.5	22	85.7	2868	
High-Risk Sex - prior 3 mo.	UAI with ≥1 male partner	86.2	29	66.1	3356	*
	UAI with ≥5 male partners	24.1	29	11.9	3356	**
	UAI with ≥1 HIV-infected or unknown serostatus male partner	34.5	29	27.5	3356	
	Transactional sex	13.8	29	7.3	3352	
	UAI after ≥5 drinks	26.1	23	23.0	2951	
	UAI while using recreational drugs	17.4	23	10.9	2950	
Prior STI		31.0	29	29.3	3356	
Post-Exposure Prophylaxis (PEP)	Heard of PEP	89.7	29	35.7	3356	**
	Used PEP	69.0	29	3.1	3354	**
Engagement in Healthcare	Contact w/any provider - prior 12 mo.	95.8	24	89.1	3087	
	Identifies a PCP	75.0	24	81.8	3087	
Communication with PCP	PCP aware of UAI behaviors^4^	58.8	17	38.0	1648	
	Comfortable discussing same-sex behaviors w/PCP	83.3	18	61.3	2517	
	Discussed ways to protect against HIV w/PCP	66.7	18	43.6	2516	*
Psychosocial Factors	History of depression	21.7	23	25.3	2963	
	Positive screen - depression (CES-D)	22.7	22	25.5	2933	
	Ever treated for drug or alcohol abuse	0	23	4.9	2946	
	Positive screen - alcohol abuse (CAGE)	4.4	23	16.4	2941	

UAI = unprotected anal intercourse; STI = sexually transmitted infection; PCP = primary care provider; CES-D = Center for Epidemiologic Studies Depression Scale; CAGE = 4-question screen for alcohol abuse.

* P≤0.05,

** P≤0.01 using test for difference between groups: t-test for continuous variables, chi-squared test or Fisher's exact test (when cell sizes are small) for categorical variables.

1 Total number of participants responding to each question.

2 Self-perceived risk of HIV acquisition = scale from 1 (no risk) to 10 (extreme risk).

3 Interested in using PrEP = likely or extremely likely to use PrEP.

4 Among participants indicating UAI in the prior 3 months.

In multivariable analysis, PrEP users were at greater odds of having had sex with men and women compared to sex with men only (OR 4.76; CI 1.40–16.2; p = 0.01), having had UAI with at least one male partner in the prior 3 months versus no UAI (OR 3.62; CI 1.00–13.1; p = 0.05), being aware of PEP compared to not being aware (OR 7.46; CI 1.52–36.6; p = 0.01), and being PEP experienced (OR 34.2; CI 13.3–88.4; p<0.0001) (Table 5).

**Table 5:** Participant Characteristics associated with Prior Use of Pre-Exposure Prophylaxis (n = 29) One Month after Publication of Efficacy Results (the iPrEx Study).

		Have Used PrEP					
		Bivariate OR	95% CI	P	Multivariable OR	95% CI	P
Gender of Sex Partners - prior 3 mo.	Men only	1.00	1.04–7.36	0.04	1.00	-	-
	Men and Women	2.77			4.76	1.40–16.2	0.01
High-Risk Sex - prior 3 mo.	UAI with ≥1 male partner	3.21	1.11–9.24	0.03	3.62	1.00–13.1	0.05
	No UAI with male partner	1.00	-	-	1.00	-	-
Post-exposure Prophylaxis (PEP)	Aware of PEP	15.6	4.71–51.6	<0.0001	7.46	1.52–36.6	0.01
	Not aware of PEP	1.00	-	-	1.00	-	-
	Prior PEP use	5.21	3.63–7.49	<0.0001	34.2	13.3–88.4	<0.0001
	No prior PEP use	1.00	-	-	1.00	-	-

OR = odds ratio; CI = Confidence Interval; P = level of significance; UAI = unprotected anal intercourse.

Variables that were not statistically significant in bivariate analyses and were not entered into the multivariable model include: age; self-perceived risk of HIV acquisition; self-identified sexual orientation; monogamous status; race/ethnicity; educational attainment; employment status; annual income; health insurance coverage; UAI with ≥1 male partner who is HIV-infected or of unknown serostatus, transactional sex, UAI after ≥5 drinks, and UAI while using recreational drugs - each over the prior 3 months; identification of a primary care provider (PCP); among participants indicating UAI in prior 3 months, having a PCP who is aware of UAI behaviors; comfort discussing same-sex behaviors with PCP; having discussed ways to protect against HIV acquisition with PCP; diagnostic history of clinical depression; positive screen for depressive symptoms (Center for Epidemiologic Studies Scale); positive screen for alcohol abuse (4-item CAGE questionnaire); and prior treatment for drug or alcohol abuse. Variables that were not statistically significant in multivariable analyses include: number of male partners for unprotected anal intercourse (UAI) and UAI with ≥5 male partners - each in the prior 3 months.

## Discussion

The successful demonstration of PrEP efficacy [2] may offer a new way to curtail the increasing number of new HIV infections among MSM in the U.S. [1] and around the world [26], [27]. Increasingly, MSM are meeting partners and obtaining health information through the internet [15], [28], [29]. The current study of MSM engaged in online social networking showed that there was an increase in PrEP awareness among a sample interviewed after the publication of the iPrEx results compared with a sample interviewed before iPrEx. Although the current study demonstrated that knowledge of PrEP immediately after iPrEx was limited overall (19%), this is not surprising as the diffusion of new knowledge clearly takes time. However, once chemoprophylaxis was described to the men, interest in PrEP use was high.

The majority of the men sampled on this online sexual network were highly educated and affluent; these demographic characteristics are consistent with those that have been previously associated with increased PrEP awareness [7]. These men are likely to represent a relatively knowledgeable subset of U.S. MSM regarding new prevention findings, so studies to assess PrEP awareness among representative samples of MSM are still needed. In this study, men who were not familiar with PEP and those who only had male partners were less likely to know about PrEP, so strategies to increase PrEP uptake for this population of MSM will need to be focused on providing information and education. MSM who were older, who reported recent UAI with a man and/or perceived themselves to be at increased risk of HIV acquisition more often indicated interest in using PrEP. This suggests that some MSM who may benefit from PrEP might be open to using it as a protective intervention. In this group, efforts should be focused on facilitating access to healthcare providers who can help individuals make informed decisions regarding PrEP use. For MSM who are younger, as well as those who may misperceive or deny their risks, education regarding risk assessment should be stressed as this will not only increase identification of those MSM who may benefit from PrEP, but could also increase utilization of HIV and STI testing.

MSM who reported prior knowledge of PEP were at 30-fold greater odds of having heard about PrEP but at nearly half the odds of expressing interest in using it, yet MSM who had used PEP were more likely to report PrEP use. A possible explanation for this paradox is that MSM who had heard about PEP, but had not used it, may have concerns about chemoprophylaxis in general, whereas those who had actually used PEP were more willing to try a similar intervention. Further studies to understand how perceptions and experiences with PEP affect attitudes and decisions regarding PrEP are needed.

The current study found minimal evidence of immediate uptake of PrEP in this community despite the fact that a majority of MSM were interested in using PrEP. It is understandable that there was limited PrEP use given the fact that the iPrEx results were released only a month prior to the second survey and implementation of medical innovations do not occur in that time frame. However, this study suggests that additional impediments to PrEP uptake may exist, as nearly 1 in 5 MSM surveyed in this study had heard of PrEP, but less than 1 in 100 had taken it. Unclear payment mechanisms could be among these barriers given estimated costs of PrEP use of $10 000 per person annually [30]. Issues related to patient-provider communication could also limit PrEP provision, as a substantial proportion of at-risk MSM in this study had not discussed risky sexual behaviors or ways to protect against HIV acquisition with a primary care provider. Therefore, it is important to improve training of providers so they become comfortable discussing MSM sexuality in an effort to identify MSM who would be most likely to benefit from PrEP. Clinicians may also be cautious about prescribing PrEP due to concerns regarding potential unintended consequences of PrEP use [3], such as toxicities, risk compensation [31], or development of drug resistance [32]. A cross-sectional survey of generalist physicians and HIV specialists in Massachusetts showed that after iPrEx, nearly all providers (92%) were aware of oral PrEP and most (76%) would be willing to prescribe it to high-risk MSM based on the results of the iPrEx study, though data to suggest the aforementioned consequences could dissuade them [Mayer et al., unpublished data]. It will be important to provide physicians with accurate data on the risks and benefits of PrEP and tools to help them communicate this information to MSM so they can share in evidence-based decision making regarding PrEP prescribing. Further studies are needed to understand facilitators and barriers to implementing this new intervention.

This study has several limitations. The most notable is that data was drawn from two different samples, using two different recruitment methods (i.e., inviting the most active users pre-iPrEx versus all members of this network post-iPrEx), resulting in different sample sizes. As such, the observed increase in PrEP awareness could potentially reflect sampling differences and not a true increase in knowledge. However, the study samples were similar with respect to variables that have previously been associated with greater PrEP awareness, such as level of PEP knowledge, education and income [7], so a bias towards increased awareness post-iPrEx is unlikely. Additional limitations suggest that participants are not representative of at-risk MSM in general. First, while the number of participants in this study was high, there was a low response rate overall (22.2% pre-iPrEx and 27.3% post-iPrEx). Other studies utilizing internet recruitment of MSM have reported rates of participation ranging from 5%–61% [33], [34], [35], [36]. Reassuringly, participant demographics in this study sample were comparable to those found in other recent studies involving members of the same online network [37], [38], making it more likely that the study sample accurately represents the network's demographics. Second, study completion rates were modest with only two-thirds of participants responding to all survey questions. However, the pattern of missing data was most consistent with non-completion of surveys because of fatigue [39], and would therefore be unlikely to bias the results of the PrEP-related outcomes asked early in the survey. Third, participants were recruited online. Yet this limitation also provides a strength in that previous studies have demonstrated that online data collection has the potential to limit social desirability bias and result in more honest and accurate responses [40], [41]. Finally, the mean age of participants was 40 years and >80% were white, so they are not representative of some of the highest risk groups, such as young black and Latino/Hispanic MSM [1]. However, nearly 40% of new infections among U.S. MSM occur in whites [1], so the study findings are likely to be relevant for a substantial population of at-risk MSM.

The findings of modest awareness, substantial interest, and minimal use of PrEP among MSM using a popular social networking website immediately after publication of iPrEx results suggests that informational campaigns are needed to raise awareness of PrEP and facilitate dialogues among at-risk MSM and their providers. As the internet may provide an effective means for disseminating risk reduction campaigns among at-risk MSM [42], [43], [44], web-based informational programs about PrEP that are targeted to this population are warranted. In addition, prospective studies are needed to understand MSM- and provider-related barriers to discussing HIV risk behaviors and initiating PrEP in the clinical setting. As efforts continue to focus on the use of biomedical interventions for HIV prevention, translation of PrEP interest into effective use of chemoprophylaxis remains a priority among strategies to decrease the rate of new HIV infections among high-risk populations.
